# Why Do Coaches Get Angry? Exploring the Relationship Between Causes of Anger and Anger Expression Styles

**DOI:** 10.3390/bs16091532

**Published:** 2026-08-31

**Authors:** Oksook Choi, Seyun Park, Geumran Seo

**Affiliations:** 1 Department of Sport Science, Chungnam National University, Daejeon 34134, Republic of Korea; choioaksuk@gmail.com (O.C.); seyunpark@cnu.ac.kr (S.P.); 2 Institute of Educational Science, Jeju National University, Jeju 63243, Republic of Korea

**Keywords:** sport anger, sport coaching, anger expression, types of anger

## Abstract

This study aimed to investigate the causes of anger experienced by coaches and to examine the relationships between these causes and different forms of anger expression. A mixed-methods design was employed. First, in-depth interviews were conducted with 10 sports coaches to identify the antecedents of coaches’ anger. Based on these findings, a pilot study involving 44 sports coaches was conducted to develop a questionnaire assessing the causes of coaches’ anger. Subsequently, a quantitative survey was administered to 267 coaches to examine the associations between the causes of anger and anger expression. The results indicated that the primary causes of coaches’ anger were Athletes’ Attitudes, External Circumstances, and Competition Situations. Compared with individual-sport coaches, team-sport coaches were more likely to perceive Athletes’ Attitudes and Competition Situations as major causes of anger. The results of the exploratory structural equation modeling (ESEM) structural model indicated that Competition Situations was negatively associated with outward expression of anger (Anger-Out) but positively associated with active self-regulation (Anger-Control) and internal suppression of anger (Anger-In). External Circumstances was positively associated with Anger-Control, whereas Athletes’ Attitudes was primarily associated with Anger-In. These findings suggest that the relationships among Anger-Out, Anger-In, and Anger-Control vary depending on the underlying causes of anger. Overall, the findings indicate that coaches’ anger is not merely a matter of individual disposition but an emotional response shaped by the complex interplay among coach–athlete relationships, competitive situations, and external factors within the sport environment.

## 1. Introduction

Anger constitutes a fundamental human emotion (Ekman, 1992), characterized by a range of intensities from mild irritation to intense fury (Spielberger, 1999). In sporting settings, anger is particularly prevalent and has been identified as one of the emotions most frequently observed among coaches (Keegan et al., 2009; Kerr & Stirling, 2012). Beyond being a basic affective state, anger is a social emotion that often arises in response to others’ behavior (Averill, 2001). Because anger is triggered by an individual’s cognitive interpretation and attribution of meaning to an event, its underlying causes have been explained from diverse theoretical perspectives (Beck, 2002; Ryoo & Chae, 2007; Seo, 2004). From a cognitive-emotional perspective, anger arises from the frustration of cognitive expectations, and its intensity increases as these expectations are more strongly thwarted (A. R. Lee, 2013). Consequently, the primary triggers of anger typically include perceived injustice, exposure to aggression or unfair treatment, and the obstruction of goal-oriented behaviors (Spielberger, 1999).

Anger is broadly categorized into trait anger and state anger. Trait anger refers to a long-term disposition characterized by a strong tendency to perceive situations as anger-provoking and to experience state anger more frequently, whereas state anger refers to a relatively temporary emotional experience (Spielberger, 1999). Considering the dynamic nature of sports contexts, the present study focuses primarily on state anger, conceptualized as discrete anger “episodes”—based on the view that anger arises from interactions between individuals and their environments (Beal et al., 2006). This perspective is particularly well-suited to exploring anger arising within coach–athlete relationships, where interactions are frequent and dynamic in sport settings.

While anger is an emotional state accompanied by physiological responses, an individual’s coping mechanism for this emotion manifests as anger expression (Goh & Hyun, 2009; Kim & Hong, 2008). Generally, anger expression is classified into three types: Anger-In, Anger-Out, and Anger-Control. Specifically, “Anger-In” refers to the tendency to suppress anger outwardly despite feeling angry, often avoiding others, remaining silent, and inwardly criticizing the target. Conversely, “Anger-Out” involves expressing anger toward individuals or objects through physical actions, criticism, swearing, verbal abuse, or extreme insults. In contrast, “Anger-Control” refers to efforts to manage and regulate anger, communicating angry feelings verbally in a non-aggressive manner while respecting the rights and emotions of others (W. H. Lee et al., 2006; Seo, 2004; Seo et al., 2004; Spielberger & Rickman, 1988).

While appropriate expression of anger facilitates problem-solving related to the causes of anger and flexible adaptation to situations, excessive expression and unconditional suppression of anger negatively affect individuals’ physical and psychological health (Cho & So, 2010; Seong & Choi, 2016). Notably, anger frequently arises in organizations characterized by high levels of interaction, where individuals in higher-status positions generally express anger more often (Gibson & Schroeder, 2002; S. K. Lee & Chang, 2014; Sloan, 2004).

Given the frequent occurrence of anger in sports settings, there is a growing emphasis on systematically understanding its functions and roles (Seong & Choi, 2016; Woodman et al., 2009). Previous research indicates that an individual’s expression of anger within an organization can lead subordinates to experience negative emotions through emotional contagion, fear, and anxiety (Seong & Choi, 2016). Similarly, coaches’ expression of anger in sports contexts directly affects athletes. Behaviors such as venting, attacking, and bottling up emotions—collectively known as anger-out and anger-in behaviors—further exacerbate anger directed towards others and increase levels of arousal and agitation; consequently, these behaviors are generally regarded as maladaptive forms of anger expression (Kuppens et al., 2004; Seong & Choi, 2016; Van Coillie et al., 2006). Such maladaptive “Anger-Out” and “Anger-In” behaviors augment the cognitive accessibility of anger-related thoughts and emotions, and provoke anxiety and fear concerning physiological sensations, ultimately culminating in adverse outcomes (Chon, 1996; Zaitsoff et al., 2002).

Nevertheless, the expression of anger by coaches—who occupy positions of authority relative to athletes within sports settings—occurs frequently. Coaches play a crucial role in transmitting emotions, thereby affecting individual athletes’ moods and the overall team environment through emotional contagion (Fransen et al., 2015). Previous research has demonstrated that coaches’ expression of anger can directly trigger an athlete’s experience of anger (S. M. Lee & Chung, 2020). Furthermore, coach anger in sports has been shown to exert either positive or negative effects on athletes’ emotions, which subsequently impact team cohesion and athletic performance (Al-Yaaribi et al., 2018). Accordingly, previous studies have examined coaches’ anger expression, anger control, and coping strategies in competition and training contexts, particularly in relation to performance enhancement (H. Choi, 2019; S. M. Lee & Chung, 2020; Y. E. Lee & Chang, 2021; S. M. Lee, 2016).

However, research on anger in sport has primarily focused on athletes; therefore, relatively little attention has been paid to understanding anger from coaches’ own perspectives. Coaches experience considerable stress as they must manage team and athletes’ performance while also serving as organizational leaders (J. Lee, 2011). Indeed, coaches are subjected to multiple performance-related, organizational, and personal stressors that can threaten their mental health and well-being (Frost et al., 2024; S. Kang & Lee, 2025). The performance-driven nature of sports not only places coaches under immense pressure to achieve top results but also exposes them to occupational insecurity contingent on performance outcomes (Bing & Jang, 2011; S. N. Kang & Yu, 2013). In such stressful environments, coaches may be more likely to display anger and coercive behaviors (Y. H. Choi & Kim, 2020), while also experiencing physical symptoms and psychological instability (E. J. Lee et al., 2022; K. Lee et al., 2019; K. Lee & Lee, 2017a, 2017b; Maslach et al., 2001; Thelwell et al., 2008a). Despite these practical challenges coaches face, a notable gap remains in the sports-related anger literature (Davis & Davis, 2016; Frost et al., 2024).

Therefore, this study aimed to identify the causes of sports coaches’ anger and examine how these causes relate to different anger expression styles, including “Anger-Out”, “Anger-In”, and “Anger Control”. By integrating qualitative exploration and quantitative analysis, this study sought to develop an empirically grounded understanding of coach anger and to provide evidence that may inform coach education, emotional regulation training, and organizational support systems in sport contexts. The research questions were as follows:

1. What are the causes of anger experienced by sports coaches in coaching contexts?
2. How do the causes of coach anger and anger expression styles differ according to coaches’ characteristics?
3. How are the causes of coach anger associated with anger expression styles, including anger-out, anger-in, and anger control?

## 2. Methods

### 2.1. Research Procedure

This study utilized an exploratory sequential mixed-method design (Creswell & Plano Clark, 2011). In the initial qualitative phase, in-depth interviews were conducted to investigate the underlying causes of coaches’ anger. Based on the qualitative findings, a quantitative survey was subsequently administered to examine variations in accordance with coaches’ characteristics and to analyze the relationships between causes of anger and types of anger expression.

### 2.2. Participants

#### 2.2.1. Interview Participants

To explore the causes of coaches’ anger, 10 coaches were selected using purposive sampling. During the participant selection process, the selection criteria were developed in consultation with an expert panel (comprising two sport psychology professors, one sport psychology counselor, and a researcher with a PhD in sport sociology), all of whom had extensive experience in scale development or qualitative research.

The criteria considered the sport type (individual or team sports) and the athletes’ age and competitive level (elementary, middle and high school, university, semi-professional, professional, and national level). Coaches who could provide the most relevant and in-depth answers to the research questions were ultimately chosen.

This study was approved by the university’s Institutional Review Board. To protect participants’ ethical protection, the purpose, content, procedures, and confidentiality of the study were thoroughly explained before the interviews, and informed consent was obtained. The research participants for the in-depth interviews are listed in Table 1.

The participants in the in-depth interviews had an average age of 40.9 years, including seven males and three females. Their coaching experience varied from 2 to 29 years. A total of 10 coaches took part, evenly split between individual and team sports. They were connected to elementary, middle, and high schools, universities, semi-professional, professional, and national teams.

#### 2.2.2. Survey Participants

The quantitative phase of the study was conducted using a survey to examine the causes of coaches’ anger and their anger expression styles. To develop a questionnaire about the causes of anger, because no existing instrument was available to measure this construct, a pilot test was conducted with 44 coaches (21 males, 23 females; 50% in their 30s).

To explore the causes of anger and how coaches express different types of anger based on their characteristics, participants were purposively recruited from a target group of active coaches from elementary, middle and high schools, universities, semi-professional teams, professional teams, and national teams nationwide. The researcher contacted potential participants by phone, message, and face-to-face interviews, and an online survey (Google Forms) was distributed through social networking services (SNS) to those who agreed to participate. As a result, 275 responses were collected; after removing 8 incomplete or invalid responses, data from 267 participants were analyzed. The characteristics of the final selected participants are presented in Table 2.

Regarding the gender of the research participants, 170 were male (63.7%) and 97 were female (36.3%). The most common age groups were 30 to under 39 years (105 participants, 39.3%) and 40 to under 49 years (102 participants, 38.2%). In terms of coaching experience, coaches with less than 5 years (78 participants, 29.2%) and 5 to under 9 years (65 participants, 24.3%) made up the largest groups, totaling over 50%. Coaches with diverse experience levels, including those with 20 or more years (25 participants, 9.4%), also participated. Regarding sport type, 165 participants (61.8%) engaged in individual sports, while 102 (38.2%) took part in team sports. The largest affiliation group was elementary, middle, and high schools with 142 participants (53.2%), followed by coaches from college (45 participants, 16.9%), semi-professional teams (43 participants, 16.1%), professional teams (20 participants, 7.5%), and national teams (17 participants, 6.4%). The gender of the coached athletes was males (93 participants, 34.8%), females (93 participants, 34.8%), and mixed-gender groups (81 participants, 30.3%).

### 2.3. Data Collection

#### 2.3.1. Interview

The interviews were conducted using both semi-structured and unstructured questioning techniques. Each participant took part in two interviews, each lasting about one hour. The process continued until data saturation was reached, indicating that no additional themes or substantively new information emerged. For example, the researcher began with a broad question, such as “Have you ever felt anger during a coaching situation?”, followed by semi-structured questions like “In what situations do you feel the most anger?” To gather more detailed and comprehensive responses, unstructured follow-up questions were added throughout the interviews. The scope and content of the interviews are outlined as follows Table 3.

#### 2.3.2. Causes of Coaches’ Anger Questionnaire

To assess the causes of coaches’ anger quantitatively, preliminary survey items were developed based on the findings from in-depth interviews. The preliminary survey consisted of 20 items rated on a 4-point Likert scale (ranging from 1: strongly disagree to 4: strongly agree) and included three subfactors, based on the types of coaches’ anger causes identified in the in-depth interviews: Athletes’ Attitudes, Competition Situations, and External Circumstances.

For the pilot study, an exploratory factor analysis (EFA) was conducted on the pilot sample (*n* = 44, 21 males, 47.7%) to provide preliminary item purification and an initial exploration of the factor structure of the Causes of Coaches’ Anger Questionnaire, rather than for definitive validation. As a result of conducting an EFA, the Kaiser-Meyer-Olkin (KMO) measure for the preliminary survey items was 0.708, and Bartlett’s test of sphericity was significant (p < 0.001), indicating suitability for factor analysis. Factor extraction was analyzed using maximum likelihood, and factor rotation was performed using direct oblimin, accounting for correlations among factors.

The internal consistency (Cronbach’s α) of the preliminary items was 0.878. As a result of consultations with researchers, the three factors in the preliminary survey items were appropriately defined as athletes’ attitudes, external circumstances, and competition situations, respectively. Two items (8 and 13) in the competition situations domain were related to athletes’ attitudes, making them heterogeneous (e.g., “I get angry when an athlete expresses dissatisfaction”). However, due to the small sample size of the pilot study, it was decided to reconsider the treatment of these two items in the main study.

In the main study, consistent with the pilot study, items related to athletes’ attitudes (Items 8 and 13) emerged within the cluster of items related to competition situations. In this regard, following a theoretical review through researcher and expert meetings, it was decided to ultimately remove these two items, as they were not directly related to competition situations. To maximize the questionnaire’s structural validity within a limited sample (*N* = 267), an EFA was initially conducted on the entire sample to explore the factor structure. Subsequently, an exploratory structural equation modeling (ESEM) analysis was conducted as a preliminary examination of the factor structure using the weighted least squares mean- and variance-adjusted (WLSMV). ESEM was selected to obtain an interpretable multidimensional solution while freely estimating all cross-loadings. This approach helped reduce the potential risk of overfitting arising from the use of the same sample and provided a preliminary assessment of the stability of the factor structure. The results of the EFA and ESEM for the 18 items finally selected are presented in Table 4.

The KMO was measured as 0.918, and Bartlett’s test of sphericity was significant (p < 0.001), indicating that the data were suitable for factor analysis. A scree plot revealed a sharp leveling off eigenvalue after the third factor; therefore, a three-factor solution was deemed appropriate and extracted. Items with a factor loading of 0.30 or greater are generally considered acceptable (Crocker & Algina, 1986). In the present study, the factor loadings ranged from 0.463 to 0.844 for the 13 items of Factor 1, 0.364 to 0.940 for the 3 items of Factor 2, and 0.577 to 0.782 for the 2 items of Factor 3. No cross-loadings were observed, which are defined as an item loading at 0.30 or higher on two or more factors with a difference in loadings of less than 0.10 across those factors (Costello & Osborne, 2005). The ESEM results showed that the global goodness-of-fit indices of the 3-factor ESEM measurement model in this study were *χ*^2^ (102) = 106.536, CFI = 0.999, TLI = 0.999, RMSEA = 0.013 (90% CI: 0.000, 0.035), and SRMR = 0.041, indicating that the acceptance criteria were generally satisfied. Factor loadings indicated that all but one item exceeded the commonly accepted threshold of 0.40 and supported the three-factor structure. The remaining item showed a relatively low standardized factor loading of 0.317. However, the item reflects a core aspect of the factor identified in previous research and the theoretical model, and its cross-loadings on the other factors were not substantial. Therefore, rather than deleting the item solely on the basis of statistical criteria, we retained it by considering its content validity and theoretical relevance in combination. Following a review by the researchers and experts to ensure the appropriateness of the item content, the three factors were named Athlete Attitude (Factor 1), External Circumstances (Factor 2), and Competition Situations (Factor 3).

The internal consistency (Cronbach’s α) for the overall causes of anger scale was 0.917, with subscale reliabilities of 0.918 for Athlete Attitude, 0.715 for External Circumstances, and 0.681 for Competition Situations, demonstrating an acceptable level of reliability.

#### 2.3.3. Korean STAXI

To assess the types of anger expression among coaches, this study used the anger expression subscale of the Korean State-Trait Anger Expression Inventory (Korean STAXI). The original STAXI was developed by Spielberger (1999) and subsequently adapted, modified, and standardized for the Korean cultural and linguistic context by Chon (1996) and Chon et al. (1998). This subscale consists of three factors: Anger-Out, Anger-Control, and Anger-In. It comprises 24 items (eight per factor), rated on a 4-point Likert scale from 1 (not at all) to 4 (very much so). This anger expression scale has been previously utilized in a study involving athletes (S. M. Lee, 2016). In a previous study involving athletes (S. M. Lee, 2016), the internal consistencies (Cronbach’s α) were 0.863 for Anger-Out, 0.817 for Anger-Control, and 0.766 for Anger-In. In the current study, the Cronbach’s α for the overall anger expression scale was 0.724, with subscale reliabilities of 0.845 for Anger-Out, 0.839 for Anger-Control, and 0.810 for Anger-In, indicating a high level of internal consistency.

### 2.4. Data Analysis

The in-depth interviews were transcribed and categorized into major typologies through a systematic process of open coding, axial coding, and selective coding. The categorized outcomes were subsequently synthesized through expert consensus to develop the questionnaire items.

The qualitative data were analyzed by three authors. Each author independently reviewed the interview transcripts and generated initial open codes. The coding results were then compared and discussed collaboratively, and any discrepancies were resolved through repeated discussion, peer debriefing, and expert consultation until consensus was reached. Through axial coding, the initial codes were grouped into conceptually related categories, which were then refined and integrated into final themes through selective coding. For example, codes such as “lying,” “lack of basic manners,” “poor self-management,” and “avoiding communication” were grouped into categories such as character issues, poor self-management, and lack of respect for the coach, and were ultimately integrated into the broader theme of athletes’ attitudes.

Survey data regarding the coaches’ causes of anger were subjected to descriptive statistical analysis to assess data normality and participant characteristics. In the initial stage of developing a questionnaire on the causes of anger, this study used the full sample to maintain adequate statistical power within the constraints of a limited sample size. Specifically, an EFA was first conducted to explore the underlying factor structure. Subsequently, an ESEM approach was employed to rigorously re-evaluate the fit of the measurement model, which was estimated using the WLSMV estimator implemented in the lavaan package in R version 4.6.1. This allowed for a precise assessment of the factor structure by freely estimating all cross-loadings, thereby avoiding the restrictive constraints of traditional measurement models.

Independent samples *t*-tests and one-way analyses of variance (ANOVAs) were conducted to explore whether the causes of anger and anger expression styles differed according to coaches’ demographic and professional characteristics. Specifically, coaches’ characteristics were analyzed using *t*-tests and Cohen’s *d* for coaches’ gender (male/female) and types of sports (individual/team sports), while age (under 30, 31–40, 41–49, 50 and older years), coaching experience (under 5, 5–9, 10–14, 15–19, 20 years and more), and athletes’ gender (male/female/mixed) were analyzed using one-way ANOVAs and Scheffé post hoc test.

To test the predictive validity of the developed scale, we avoided the inconsistency of using observed composite scores by implementing a comprehensive ESEM-within-CFA framework (Morin et al., 2020). To rigorously evaluate the relationships among these latent variables, types of sport were directly embedded into the structural equation model as a covariate, simultaneously adjusting for its influence across the three anger dimensions.

## 3. Results

The present study explored anger-inducing situations from the perspective of sports coaches. Accordingly, the causes of anger experienced by coaches, their types of anger expression, and the relationships between these variables were investigated, yielding the following results.

### 3.1. Causes of Anger Among Sports Coaches

The causes of anger among coaches were categorized into three main themes: Athletes’ Attitudes, External Circumstances, and Competition Situations. The specific details are presented in Table 5.

First, coaches reported experiencing anger due to factors related to athletes’ attitudes. These included character issues (e.g., lying, lack of basic manners), poor self-management, behaviors undermining team cohesion (e.g., egocentric decision-making, selfish behavior), inadequate attitudes towards athletic performance (e.g., repeated mistakes, lapses in concentration, discrepancies between expected and actual performance), and a lack of respect for the coach (e.g., avoiding communication, failing to fulfill commitments). In particular, coaches frequently identified athletes’ failure to engage in appropriate self-management as a primary cause for their anger.

*“There are cases where athletes drink and miss training, cases where they cannot participate in competitions because they fail to meet minimum academic standards, and cases where they fail to manage their weight. When I speak to them, they need to show some effort, but [when they do not]… it makes me angry.”*

(Coach A)

Athletes at the university, semi-professional, or professional level are adults who are afforded greater autonomy in managing their time than middle and high school student-athletes. This autonomy also entails personal responsibility. University student-athletes must navigate the dual demands of academics and athletics, meeting both competition eligibility criteria and minimum academic requirements. In these situations, even when coaches provided sufficient explanations about self-management, some athletes still failed to meet their daily commitments or overlooked proper self-management. Such neglect was a major cause of coaches’ anger. Additionally, coaches’ anger was often triggered by athletes’ behavior during training.

*“I get angry when they fail to do what we originally agreed they should do. [Omitted] I get angry when athletes make mistakes. I think, ‘They could have avoided that mistake, but they did it again.’ It makes me angry when they repeatedly make the exact same mistakes during training.”*

(Coach B)

*“First, ours is a team sport, so every athlete has a position. I get angrier when athletes seem to be putting in a half-hearted effort during training. From a coach’s perspective, it is obvious. You can see whether they are giving 100% or just going through the motions. I get the angriest when they are just going through the motions.”*

(Coach C)

As demonstrated in the participants’ statements above, coaches’ anger was triggered when athletes failed to execute prearranged plays, made repeated mistakes, or lacked sufficient effort and concentration during training. In general, match outcomes are among the pivotal achievement goals targeted by coaches. Within a sport setting that places considerable emphasis on victory, results function as a primary metric for evaluating coaches’ performance; consequently, coaches are likely to respond with sensitivity to athletes’ attitudes and behaviors that might relate to these outcomes. This indicates that coaches are highly sensitive not only to match outcomes but also to athletes’ attitudes, encompassing self-management, interpersonal relationships, mutual respect, and training demeanor.

Second, the causes of coaches’ anger encompassed competitive situations, such as critical moments during a match or experiencing defeat. It was confirmed that coaches experienced anger when they perceived their team to be losing or when facing an unfavorable match outcome.

*“I get angry during matches… but because there are eyes watching in a match situation, rather than protesting strongly, I speak calmly… or I just don’t say anything at all during timeouts. Then the athletes sense the atmosphere… they realize the coach is angry.”*

(Coach A)

*“Recently, what makes me the angriest during matches is when it’s obvious we’ve practiced a lot, but they show anxiety or become too obsessed with winning and losing. When I see that, I get angry without realizing it… Did we not train enough? Because… we map out all the plans. But it doesn’t go according to plan. We practice every day. We do it every day, but during the match, the pace slows down… [Omitted]”*

(Coach C)

As noted above, coaches experienced anger when athletes failed to perform to their trained abilities during actual competitions despite adequate practice, or when the team was losing or placed at a competitive disadvantage against an opponent. Notably, some coaches, aware that competitions take place in public settings under the observation of spectators and stakeholders, deliberately regulated their emotional responses by offering controlled feedback or remaining silent. Specifically, they managed their emotions by conveying their displeasure through nonverbal cues rather than overt outbursts.

Third, the causes of coaches’ anger were identified as external circumstances, including excessive parental intervention, inadequate administrative and institutional support, and perceived unfairness in refereeing decisions. This demonstrates that the external environment and institutional factors surrounding coaches are significantly related to their anger.

*“In our sport, rather than saying nice things, there are times when parents secretly cause discord behind our backs… [Omitted] Honestly, only a set number of athletes can play, and there are many times they complain, asking, ‘Why can’t my child play when they are good?’ or ‘Why do you train them like this?’ I get angry in these situations.”*

(Coach E)

*“During the COVID-19 period, a national competition was held. Clubs could just send an official document, and parents could gather and go individually, but we [school teams] had to move as a team. Being affiliated with a school meant we had to go through various procedures like business trip approvals, and during that process, they just said ‘absolutely not’ because of COVID. If they just say ‘no’ without trying to find a solution, there is nothing we can do.”*

(Coach F)

As the statements illustrate, interventions from external entities such as schools, associations, clubs, and parents are perceived as encroaching upon the coach’s authority and decision-making independence. For example, some parents expressed significant dissatisfaction with their children’s playing time and coaching techniques. Such circumstances caused coaches to feel that their coaching methods were being disrespected, which subsequently elicited anger. Additionally, coaches’ anger was often attributed to concerns regarding referees’ fairness.

*“When our athletes are facing disadvantages, I have to protest, and I get very angry at those times. Because I have to protect our athletes, I yell at the referee to overturn the decision, and it even leads to physical scuffles. After a tournament, there are times when my voice is completely hoarse.”*

(Coach B)

*“There are times when a specific referee makes calls uniquely disadvantageous to our team. Then, looking at the referee assignments, I think, ‘Ah, we’re going to have a tough match today.’ It’s the exact same situation, but they blow the whistle against our team and not the opponent… So, I get angry while protesting… and I get even more angry seeing the athletes fail to play their game because they are too busy getting angry at the referee’s decisions.”*

(Coach D)

Therefore, coaches’ anger was caused by unfair refereeing decisions, especially during matches when they believed their athletes were being unjustly disadvantaged. They often protested loudly to try to overturn the decisions, sometimes displaying this through aggressive behavior. This illustrates that coaches’ anger is not solely a product of their relationship with athletes but is a psychosocial emotion manifesting within complex relationships involving various external stakeholders (e.g., parents, institutional support, referees).

### 3.2. Causes of Anger and Types of Anger Expression According to Coach Characteristics

This study analyzed whether the causes and expressions of anger may differ depending on the characteristics of the coach. Among the characteristics of coaches, only types of sports showed significant differences in both the causes of anger and anger expression. Coaches’ gender, age, coaching experience, and athletes’ gender were not characteristics that showed differences in both the causes of anger and anger expression.

Specifically, differences in the causes of anger were found by types of sports for Athletes’ Attitudes (t = −3.039, p<0.01, d = −0.357, 95% CI [−0.606, −0.108]) and Competition Situations (t = −2.218, p < 0.05, d = −0.294, 95% CI [−0.542, −0.046]) with team sport coaches perceiving these factors as causes of anger to a greater extent than individual sport coaches. No significant differences in External Circumstances were observed across types of sports (t = −0.572, p>0.05, d = −0.072, 95% CI [−0.319, 0.175]). Anger expression showed significant differences based on types of sports. Specifically, team-sport coaches reported significantly higher levels of Anger-Out than individual-sport coaches (t = −2.851, p < 0.01, d = −0.359, 95% CI [−0.608, −0.110]). No significant differences by sport type were found for Anger-Control (t = 1.363, p>0.05, d = 0.172, 95% CI [−0.076, 0.419]) and Anger-In (t = −0.600, p>0.05, d = −0.076, 95% CI [−0.322, 0.171]).

### 3.3. Relationships Between Causes of Anger and Types of Anger Expression

Based on the relationships between coaches’ causes of anger and their types of anger expression, analyses were conducted to determine which anger-inducing factors are significantly related to specific anger expression styles.

A Pearson correlation analysis was conducted to examine the relationships among the sub-factors of the causes of anger and types of anger expression. The results are presented in Table 6.

The analysis revealed that among the types of anger expression, Anger-Out and Anger-In were positively correlated with all anger causes, whereas Anger-Control was negatively correlated with the Competition Situations factor. Specifically, Anger-Out showed the strongest positive correlation with Athletes’ Attitudes (r = 0.478, p < 0.001), followed by External Circumstances (r = 0.344, p < 0.001) and Competition Situations (r = 0.340, p < 0.001). Anger-In was most strongly correlated with External Circumstances (r = 0.450, p < 0.001), while also demonstrating positive correlations with Athletes’ Attitudes (r = 0.361, p < 0.001) and Competition Situations (r = 0.386, p < 0.001). For Anger-Control, a weak but significant negative correlation was found with Competition Situations (r = −0.181, p < 0.01), suggesting that when anger is caused by competitive situations, coaches’ ability to control their anger diminishes.

These findings indicate that all causes of anger are associated with both expressing and suppressing anger (Anger-Out and Anger-In); however, Anger-Out is more closely related to athletes’ attitudes, whereas Anger-In is more closely associated with external circumstances. Conversely, Anger-Control was negatively correlated solely with competitive situations, implying that anger regulation may become particularly difficult when anger is induced during matches.

The structural relations among the latent constructs were examined while controlling for the covariate, types of sports. The results of the standardized path coefficients and their significance levels are presented in Table 7.

The overall fit of the model, which incorporated a highly complex multidimensional latent structure comprising 42 observed variables (18 items for predictors, 24 items for criteria) alongside a covariate (types of sports), was evaluated using the WLSMV estimator. An oblique target rotation was applied to the factors. The model yielded *χ*^2^ (813) = 1604.336, CFI = 0.910, TLI = 0.900, RMSEA = 0.060 (90% CI [0.056, 0.065]), and SRMR = 0.087. Overall, the model showed an adequate level of fit, with the CFI and TLI reaching approximately 0.90 and the RMSEA indicating an acceptable degree of approximate fit. However, the SRMR was slightly above the commonly used criterion of 0.08, suggesting that the model fit was acceptable but not unequivocally good.

Regarding Anger-Out, competition situations were significantly and negatively associated with Anger-Out (β = −0.381, p < 0.001). The model explained 13.8% of the variance in Anger-Out ($R^{2}$ = 0.138, p < 0.01). Athletes’ attitudes and external circumstances were not significantly associated with Anger-Out. For Anger-Control, both external circumstances and competition situations were significantly and positively associated with Anger-Control (β = 0.445, p < 0.001 and β = 0.458, p < 0.001, respectively). The model accounted for 51.1% of the variance in Anger-Control ($R^{2}$ = 0.511, p < 0.001). Regarding Anger-In, athletes’ attitudes (β = 0.318, p < 0.001) and competition situations (β = 0.368, p < 0.001) were significantly and positively associated with Anger-In, whereas external circumstances were not significantly associated with Anger-In. The covariate, types of sports, was also significantly and positively associated with Anger-In (β = 0.173, p < 0.01). The model explained 39.3% of the variance for Anger-In ($R^{2}$ = 0.393, p < 0.001).

## 4. Discussion

This study aimed to explore coaches’ anger in coaching situations by examining the connections between recurring causes of anger and anger expression styles, rather than viewing anger only as a reflection of individual traits or skills. Through in-depth interviews, the study initially identified and categorized the causes of anger experienced by coaches. Building on these qualitative findings, the newly developed Causes of Anger Questionnaire and the Korean version of the STAXI were used to examine differences in causes of anger and anger expression styles based on coaches’ characteristics, and to analyze the relationships among these variables.

### 4.1. Understanding the Causes of Anger Among Sports Coaches

Anger is a prevalent emotion in sport and is among the most frequently observed emotions among coaches. In this investigation, the causes of coaches’ anger were identified as athlete attitude, external circumstances, and competition situations. Firstly, concerning the causes of coaches’ anger, “athlete attitude” was linked to athletes’ dishonesty, disrespect, inadequate self-management, and behaviors that undermine the team atmosphere. Such behaviors contravene the standards of mutual respect and role fulfillment expected by coaches from athletes; therefore, athlete attitude was found to be a cause to which coaches responded with the greatest sensitivity. Coaches serve as pivotal agents in shaping athletes’ behavioral standards and value systems and, in certain instances, assist in their development as social members. In this context, when athletes’ attitudes fail to meet coaches’ expectations, these attitudes may be perceived as impediments to athlete development and team cohesion, subsequently provoking feelings of anger (Côté & Gilbert, 2009; Harwood & Knight, 2016).

Secondly, “external circumstances” as a cause of coaches’ anger encompass refereeing decisions, parental involvement, and administrative issues. These factors are often difficult for coaches to control directly, and anger tends to emerge particularly when coaches’ authority and autonomy are compromised by external stakeholders. In sports settings, coaches are positioned at the intersection of athletes’ performance demands and the expectations of multiple stakeholders, including parents, referees, and sports associations. Notably, coaching involves emotional labor, as coaches are required to regulate and express emotions in professional settings; these emotional demands have been linked to burnout (Y. H. Lee et al., 2015). Consequently, coaches’ anger resulting from external circumstances should not be viewed solely as an individual emotional response. Instead, it may reflect the emotional burden faced by contemporary sports coaches, who are compelled to manage professional responsibilities and external pressures despite having limited control over these conditions.

Third, “competition situations” were identified as a cause of coaches’ anger when athletes failed to perform to their expected level during competition or when the team lost a game. Competition situations are directly related to coaches’ role performance because they are often perceived as visible indicators of the effectiveness of coaches’ instructional strategies and are linked to evaluations of their professional competence and expertise (Côté & Gilbert, 2009). In sports contexts where a win-at-all-costs culture remains prevalent, competition results often serve as a critical indicator of coaches’ ability. Accordingly, public evaluation and pressure on coaches may generate a context in which anger is directed toward athletes. However, some coaches might also deliberately express anger towards athletes or referees during competition, believing that such actions can improve athletes’ performance (Davis & Davis, 2016). In this regard, the causes of coaches’ anger should not be treated as fixed or universal; rather, they may vary depending on individual dispositions, situational and structural factors, and coaches’ emotion regulation capacities.

### 4.2. Differences in Causes of Anger and Anger Expression by Coach Characteristics

The present study demonstrated that the causes of coaches’ anger vary significantly by types of sports. Specifically, compared with coaches in individual sports, coaches in team sports regarded ‘athletes’ attitudes’ and ‘competition situations’ as considerably more prominent catalysts of anger. Additionally, coaches in team sports showed higher levels of ‘Anger-Out’ behaviors. Overall, these findings suggest that coaches involved in team sports not only experience increased anger in response to athlete behaviors and competition situations but are also more inclined to express this anger outwardly. The findings from in-depth interviews with coaches in this study, which mentioned athletes’ behaviors that undermine team cohesion as a cause of anger induction, are consistent with these results. This phenomenon is likely attributable to the intrinsic characteristics of team sports, where an individual athlete’s disciplinary infractions or performance failures can directly threaten collective cohesion (Eys et al., 2015). Because reduced cohesion can undermine team performance, coaches may interpret such situations as threats to team functioning and their sense of control, thereby increasing the likelihood of experiencing and outwardly expressing anger (Fransen et al., 2020).

### 4.3. Understanding the Relationships Between Causes of Anger and Types of Anger Expression

The bivariate correlation analysis showed that Anger-Out and Anger-In were significantly associated with all Causes of Anger subfactors, whereas Anger-Control exhibited a very small negative association with Competition Situations. These findings suggest that the Causes of Anger subfactors may have distinct relationships with different forms of anger expression. Accordingly, given the limitations of bivariate correlations, in which each pair of variables is examined separately, the present study employed exploratory structural equation modeling (ESEM), which simultaneously considers all subfactors of the Causes of Anger and anger expression constructs.

The ESEM results indicated that the directions of the associations between Competition Situations and Anger-Out and between Competition Situations and Anger-Control differed from those observed in the bivariate correlation analysis. This discrepancy between the bivariate correlations and the latent factor correlations estimated in the WLSMV-ESEM may be attributed to differences in the analytical level and estimation procedure. Specifically, bivariate correlations reflect unadjusted associations between observed subscale scores, whereas the factor correlations estimated in ESEM represent associations between latent constructs while accounting for the ordinal nature of the items, measurement error, the simultaneous estimation of all factors, and freely estimated cross-loadings. Therefore, the ESEM results provide a more comprehensive account of the relationships among the Causes of Anger and anger expression subfactors than do pairwise bivariate correlations.

The present study revealed significant pathways between the causes of coaches’ anger and the subfactors of anger expression. Specifically, Competition Situations was negatively associated with outward expression of frustration (Anger-Out) and positively associated with active self-regulation (Anger-Control) and internal suppression of anger (Anger-In). This pattern suggests that anger arising in competitive situations may be associated with a lower tendency to express frustration outwardly, alongside greater use of active self-regulation and internal suppression. Additionally, while ‘external circumstances’ increased coaches’ ‘Anger-Control’, ‘athletes’ attitudes’ were significantly associated only with ‘Anger-In’. This aligns with existing emotion research suggesting that the experience of anger can be distinguished from the way it is expressed (Averill, 2001; Spielberger, 1999).

Among the causes of coaches’ anger, competition situations emerged as a key factor that decreased maladaptive Anger-Out while simultaneously promoting both Anger-Control and Anger-In. Because the sports competition environment involves simultaneous time pressure, performance evaluation, and public exposure, coaches’ emotional regulation abilities are often put to the test (Olusoga et al., 2010; Thelwell et al., 2008a). In addition to emotional changes, previous studies have shown that coaches experience increased psychological and physiological stress responses, as well as elevated levels of unpleasant feelings and arousal during competitions (Hudson et al., 2013; Obmiński et al., 2022).

Based on these findings, it can be inferred that because coaches are highly likely to experience anger during competitions, they consciously restrain themselves from expressing it outwardly; conversely, they perceive a greater need to exert effort to suppress and control their anger. Furthermore, because sports coaches act as representatives of their organizations in the highly public environment of a stadium, they are likely to be compelled to reinforce their anger-suppression strategies in such settings (Y. H. Lee et al., 2015). In this regard, Thelwell et al. (2008b) reported instances where coaches actively strive to suppress their emotions and refrain from outwardly expressing them during competitions.

When coaches’ anger was caused by external factors rather than competition situations or athletes, their Anger-Control was still promoted. Coaching is inherently a performance-driven profession where individuals experience significant stress related to team outcomes and work–life balance. In fact, many job-related stressors reported by coaches involve time pressure, maintaining a work–life balance, providing leadership and motivation to athletes, and securing successful seasonal results to maintain job stability (Powell et al., 2022). Similarly, another study found that coaches face various occupational and social stressors—such as quitting, task-related sources, recruiting, time demands, and the pressures of being the head coach—in addition to interpersonal/personal sources, other people, outcomes of competition, and self-imposed stress (Frey, 2007). In many ways, these stressors experienced by sports coaches are similar to those encountered by professionals in other highly demanding occupations (Fletcher & Scott, 2010). Given this context, these findings suggest that coaches’ reliance on anger control in response to external circumstances is closely related to the need to simultaneously navigate relationships with outside stakeholders, their organizational position, and professional role expectations.

Athletes’ attitudes yielded a pathway affecting coaches’ Anger-In. During the coaching process, one of the most frequently experienced emotions—aside from stress-related anxiety—is anger (Kerr & Stirling, 2012; Omli & LaVoi, 2009). However, because coaches’ emotions can significantly influence athletes’ emotional regulation, coaches’ ability to lead effectively is related to their own emotional regulation skills (Hill & Davis, 2014). Evidence shows that coaches’ outward expression of anger is associated with increased athlete errors and diminished team performance, whereas positive emotional expression is linked to positive team outcomes (Van Kleef et al., 2019). This underscores the critical importance of coaches’ emotional regulation for effective coaching. In this context, although coaches frequently experience anger in response to athletes’ attitudes, they likely exert greater effort to suppress their anger to fulfill their educational responsibility for athlete development (Chung, 2004; Côté & Gilbert, 2009) and to maintain effective leadership.

## 5. Conclusions and Recommendations

The findings of this study indicate that coaches’ anger is not merely a personal disposition but also a complex emotional response shaped by coach-athlete relationships, competition situations, and external environments. Through in-depth interviews, the primary causes of coaches’ anger were identified as athletes’ attitudes, external circumstances, and competition situations. Furthermore, compared with individual sport coaches, team sport coaches reported higher levels of anger related to athletes’ attitudes and competition situations and were more likely to express anger outwardly.

Among these causes of anger, competition situations emerged as a major factor related to all anger expression styles. Specifically, structural analyses indicated that when anger was caused by competition situations, coaches exhibited reduced Anger-Out alongside increases in Anger-Control and Anger-In. Additionally, higher levels of External Circumstances were associated with higher levels of Anger-Control, whereas Athletes’ Attitudes were primarily associated with Anger-In. Ultimately, the findings demonstrate that the dynamics among Anger-Out, Anger-In, and Anger-Control differ significantly depending on the specific causes of anger, providing a more nuanced understanding of coaches’ emotions in coaching contexts.

As described above, this study examined the relationship between the causes of coaches’ anger and their patterns of anger expression, focusing on anger as one of the emotions most commonly experienced by coaches. To this end, we developed a questionnaire assessing the causes of coaches’ anger and conducted a preliminary validation study. To preliminarily examine the factor structure of the Causes of Coaches’ Anger Questionnaire, we employed ESEM, which allows all cross-loadings to be freely estimated and facilitates the identification of an interpretable multidimensional solution. This approach provided a more flexible examination of the factor structure than a model imposing zero cross-loadings. However, because the EFA and ESEM analyses were conducted using the same sample, the possibility of sample-specific overfitting cannot be ruled out. Thus, the generalizability of the questionnaire and the stability of its factor structure should be evaluated in future research using an independent sample or cross-validation.

In addition, the findings indicated the need to increase the number of items assessing the Competition Situations factor and to improve the internal consistency of this subscale. The present study also has several limitations related to its cross-sectional design, the relatively homogeneous sample used in the structural model, and the specific characteristics of the Korean coaching culture and context, which may limit the generalizability of the findings. Future research should address these limitations by recruiting larger and more diverse samples of coaches, further examining the validity and reliability of the questionnaire, and investigating the relationships among the study variables across different populations and contexts.

## Figures and Tables

**Table 1:** Characteristics of In-Depth Interview Participants.

	Gender	Age	Affiliation	Types of Sports	Coaching Experience (Years)
1	male	37	Elementary school	Individual	15
2	male	31	Middle school	Team	2
3	female	40	High school	Individual	13
4	male	43	High school	Team	18
5	male	41	College	Team	6
6	male	58	College	Team	29
7	male	37	Semi-professional	Individual	9
8	male	40	Professional	Team	3
9	female	49	National	Individual	27
10	female	33	National	Individual	2

**Table 2:** Characteristics of Survey Participants.

Coach Characteristics	Category	*N*	%
Gender	Male	170	63.7
	Female	97	36.3
Age	Under 30 years	24	9.0
	30–39 years	105	39.3
	40–49 years	102	38.2
	50 years and older	36	13.5
Coaching Experience	Under 5 years	78	29.2
	5–9 years	65	24.3
	10–14 years	53	19.9
	15–19 years	46	17.2
	20 years and over	25	9.4
Types of Sports	Individual	165	61.8
	Team	102	38.2
Affiliation	Elementary, middle and high schools	142	53.2
	College	45	16.9
	Semi-professional	43	16.1
	Professional	20	7.5
	National	17	6.4
Athletes’ Gender	Male	93	34.8
	Female	93	34.8
	Mixed	81	30.3

**Table 3:** Scope and Content of In-Depth Interviews.

Overview	Core Contents
Personal characteristics	Coach gender, coach age, coaching experience, sport type, coach affiliation, coached athletes’ gender, perceived athletes’ performance level
Coaches’ anger experiences	Anger experiences triggered by athletes in coaching situations, causes, frequency, cognitive appraisal of anger, modes of anger expression, athletes’ responses, emotions after anger

**Table 4:** Comparison of factor loadings for each item based on EFA and ESEM.

Items	EFA	ESEM	a
**Athletes’ attitudes**			0.918
I get angry when an athlete does not admit their fault.	0.844	0.813	
I get angry when an athlete acts selfishly.	0.839	0.860	
I get angry when an athlete does not observe basic manners.	0.744	0.954	
I get angry when an athlete does not concentrate during instruction.	0.715	0.767	
I get angry when an athlete repeatedly fails to remember despite multiple instructions.	0.692	0.694	
I get angry when an athlete lies or makes excuses.	0.676	0.831	
I get angry when an athlete fails to keep promises regarding their basic lifestyle and attitude.	0.605	0.718	
I get angry when an athlete participates in a competition or training lethargically.	0.596	0.708	
I get angry when an athlete fails to execute agreed-upon tactics or strategies.	0.563	0.649	
I get angry when an athlete expresses negative emotions after making a mistake.	0.552	0.548	
I get angry when I feel that an athlete is ignoring me.	0.481	0.648	
I get angry when an athlete neglects self-management.	0.478	0.651	
I get angry when an athlete fails to do what I believe they are capable of.	0.463	0.579	
**External Circumstances**			0.715
I get angry when external parties (e.g., parents, schools, associations, clubs) interfere with my coaching authority regarding a competition.	0.940	0.658	
I get angry when external parties (e.g., parents, schools, associations, clubs) interfere with training methods or the operation of the sports team.	0.724	0.491	
I get angry when a referee’s decision is unfair.	0.364	0.317	
**Competition situations**			0.681
I get angry when we are losing during a competition.	0.782	0.890	
I get angry when we lose a competition.	0.577	0.610	

**Table 5:** Causes of Anger Among Coaches.

Theme	Sub-Themes (Coded Categories)
Athletes’ Attitudes	Character issues (lying, lack of basic manners), poor self-management, behaviors undermining team cohesion (egocentric decision-making, selfish behavior), inadequate attitudes towards athletic performance (repeated mistakes, lapses in concentration, expectation-performance discrepancy), lack of respect for the coach (avoiding communication, failing to fulfill commitments), external circumstances
External Circumstances	Excessive parental intervention, inadequate operation and systems of schools and federations, issues with the fairness of refereeing decisions
Competition Situations	Crisis situations during competition, defeat in a match

**Table 6:** Correlations Between Causes of Anger and Types of Anger Expression.

		Causes of Anger		
		Athletes’ Attitudes	External Circumstances	Competition Situations
Types of Anger Expression	Anger-Out	0.478 ***	0.344 ***	0.340 ***
	Anger-Control	−0.080	−0.063	−0.181 **
	Anger-In	0.361 ***	0.450 ***	0.386 ***

** *p* < 0.01, *** *p* < 0.001.

**Table 7:** ESEM-Based Verification of Structural Path Estimates.

DV	IV	B	S.E.	Z	β	p
Anger-Out	Athletes’ Attitudes	0.004	0.031	0.122	0.009	0.903
	External Circumstances	0.03	0.035	0.857	0.075	0.391
	Competition Situations	−0.152	0.033	−4.636	−0.381	***
	Types of sports (Covariate)	−0.074	0.056	−1.315	−0.09	0.189
Anger- Control	Athletes’ Attitudes	−0.055	0.049	−1.123	−0.082	0.262
	External Circumstances	0.298	0.056	5.342	0.445	***
	Competition Situations	0.306	0.047	6.531	0.458	***
	Types of sports (Covariate)	0.04	0.092	0.435	0.029	0.663
Anger-In	Athletes’ Attitudes	0.254	0.055	4.629	0.318	***
	External Circumstances	0.089	0.061	1.46	0.111	0.144
	Competition Situations	0.294	0.056	5.275	0.368	***
	Types of sports (Covariate)	0.285	0.107	2.659	0.173	**

Note. DV: dependent variable; IV: independent variable; B: unstandardized estimate; S.E.: standard error; Z: z statistic; *β*: standardized estimate; ** *p* < 0.01; *** *p* < 0.001.

## Data Availability

The data presented in this study are available from the corresponding author upon reasonable request. Public access to the data is restricted due to privacy and ethical concerns of research participants.
